# The influence of prenatal dexamethasone administration before scheduled full-term cesarean delivery on short-term adverse neonatal outcomes: a retrospective single-center cohort study

**DOI:** 10.3389/fped.2023.1323097

**Published:** 2024-01-11

**Authors:** Jiaojiao Pei, Jiao Chen

**Affiliations:** Department of Hepatobiliary Surgery, the First Affiliated Hospital of Chengdu Medical College, Xindu District, Chengdu, Sichuan Province, China

**Keywords:** antenatal corticosteroids, planned cesarean, newborn outcomes, termination of pregnancy, retrospective cohort study

## Abstract

**Objective:**

There has been a gradual increase in the prevalence of cesarean section deliveries and more healthcare professionals are considering the prophylactic use of corticosteroids before planned full-term cesarean sections. However, the association between dexamethasone administration before full-term cesarean delivery and short-term adverse neonatal outcomes is unclear. This study analyzed the disparities in short-term adverse neonatal effects in neonates born via full-term elective cesarean delivery with or without antenatal dexamethasone treatment.

**Study design:**

This single-center retrospective cohort study involved neonates aged 37–39 weeks. The primary neonatal outcomes included various short-term adverse events, including neonatal admission to the neonatal intensive care unit, neonatal access to the special care baby unit, transient neonatal respiratory distress, respiratory distress syndrome, and the requirement of intravenous antibiotics or ventilatory support. Multiple logistic regression analysis was used to assess the association between these outcomes and dexamethasone exposure while adjusting for covariates.

**Results:**

Of the 543 neonates included in the study, 121 (22.2%) had been exposed to prenatal dexamethasone. When compared with the control group, the dexamethasone-exposed group exhibited significantly higher rates of transient neonatal respiratory distress, respiratory distress syndrome, administration of intravenous antibiotics, the need for ventilatory support, and longer duration of neonatal hospitalization (*P* < 0.05). The association between dexamethasone exposure and short-term adverse neonatal outcomes remained significant after adjusting for potential confounders (odds ratio: 12.76, 95% confidence interval: 6.9–23.62, *P* < 0.001).

**Conclusion:**

The dexamethasone-exposed group had a higher likelihood of experiencing short-term adverse outcomes when compared with non-exposed neonates, suggesting that dexamethasone may have detrimental effects on infants delivered at full term. This implies the importance of exercising caution when contemplating the use of antenatal corticosteroids.

## 1 Introduction

In recent decades, the rate of cesarean section deliveries has markedly increased worldwide, especially in high and middle-income countries (1–3). However, cesarean delivery independently contributes to the risk of neonatal respiratory complications, mainly respiratory distress syndrome and transient neonatal tachypnea (4, 5). Because the risk associated with elective cesarean delivery diminishes as gestational age progresses (6), it is recommended to postpone the procedure until the pregnancy reaches 39 weeks (7–10). However, approximately 10%–15% of women who choose cesarean section may deliver before the recommended gestational age (11). The Antenatal Steroid Trial for Full-Term Elective Cesarean Sections (12) and the Cochrane Review on the use of corticosteroids to prevent respiratory disorders in neonates after full-term elective cesarean deliveries (13) have led to widespread clinical acceptance of the use of prophylactic corticosteroids before scheduled full-term cesarean sections as a mitigation for potential neonatal respiratory system risks.

The Royal College of Obstetricians and Gynaecologists (RCOG) Green Top Guideline No. 7 (RCOG 2010) (14) recommended the prophylactic use of corticosteroids before full-term planned cesarean sections. However, this recommendation is not included in the current guidelines by the National Institute for Health and Care Excellence (NICE) for cesarean sections (NICE 2021) (15).

Dexamethasone, a synthetic glucocorticoid, is widely used to manage preterm labor and promote fetal pulmonary maturation, especially in low-resource settings (16, 17). The administration of prenatal corticosteroids promotes the maturation of the fetal pulmonary system in premature infants, thereby decreasing the occurrence of respiratory distress syndrome, the need for respiratory support, the duration of intensive care hospitalization, and the prevalence of various premature neonate complications, such as intraventricular hemorrhage, necrotizing enterocolitis, and neonatal mortality (18). The Maternal and Child Health Survey, a comprehensive investigation by the World Health Organization involving 359 institutions in 29 countries found that the prevalence of synthetic glucocorticoid administration was 54%, although some countries have rates of up to 91% (19).

The use of dexamethasone during late preterm birth is controversial, especially in low-resource settings (17). A thorough assessment was conducted to determine the efficacy of administering corticosteroids before full-term, scheduled cesarean deliveries and if it provides significant advantages while avoiding unnecessary harm. This harm is attributable to a limited understanding of the cellular and molecular mechanisms governing fetal lung maturation (20), coupled with inherent limitations of currently available markers (21). Evidence indicates that neonates exposed to prenatal corticosteroids may have a higher risk of unfavorable outcomes (22–25). Thus, the use of prenatal corticosteroids in the context of term deliveries warrants careful consideration.

## 2 Materials and methods

### 2.1 Data source and study cohort

The data used underlying this study are from the Dryad Digital Repository (https://doi.org/10.5061/dryad.g79cnp5qs). This retrospective single-center cohort study was carried out from December 2016 to February 2019 at the Professorial Unit, Department of Obstetrics and Gynecology, Colombo South Teaching Hospital University. The study population was described previously (26). The study participants were divided into the experimental and control groups. The experimental group comprised of mothers who were administered two intramuscular injections of dexamethasone (12 mg) at 12-hour intervals, commencing from one week to 24 h prior to delivery. The control group was made up of mothers who did not receive corticosteroid treatment before delivery.

This study involved maternal–fetal dyads who underwent elective cesarean sections at the gestational age of 37–39 weeks and who met the inclusion criteria. The cesarean sections analyzed in this study were categorized as elective, defined as cesarean sections scheduled in advance and not conducted under emergency circumstances. Information on cesarean section indications, such as elective factors like maternal request, large fetus, malpresentation/breech, and repeat cesarean section, was obtained from medical records. Neonatal care was provided within a single unit, ensuring uniform diagnostic and admission prerequisites.

The study cohort was subjected to exclusionary criteria, which included symptoms of severe maternal hypertension, severe fetal rhesus alloimmunization, or intrauterine infection characterized by maternal pyrexia, tachycardia, fetal distress, and meconium-stained liquor at delivery. Pregnant women who were concurrently receiving steroids for reasons unrelated to the study protocol, those with multiple gestations, those with emergency conditions necessitating mandated cesarean section, and cases with insufficient covariate data, were also excluded.

The requirement for ethical approval was waived because all data were meticulously anonymized and the study strictly adherence to the protocols and regulations established by the Dryad Digital Repository.

### 2.2 Data acquisition

Relevant patient bedside records were retrieved from archival records and subsequently transcribed onto a designated data collection template by an assistant researcher. To mitigate observer bias, a second assistant researcher not involved in developing the study protocol or in patient management, compiled the maternal demographic data, including data on maternal age, diversity of gestation, previous and current medical conditions, gestational age at the time of cesarean section, intricate surgical particulars, and postoperative complications. Moreover, data on the administration of corticosteroids to the mothers was meticulously documented. Relevant neonatal data, including birth weight and Apgar scores, were recorded. The primary outcomes under investigation included neonatal admission to the neonatal intensive care unit (NICU), assignment to the special care baby unit, transient neonatal tachypnea, respiratory distress syndrome, intravenous antibiotic administration, need for ventilatory support, and duration of neonatal hospitalization. Any of the first six above-mentioned criteria indicated short-term adverse neonatal effects.

### 2.3 Statistical analysis

First, we summarized the study cohort's baseline characteristics and categorized them based on dexamethasone exposure status. For continuous data, descriptive statistics involved the use of either the mean and standard deviation or the median and interquartile range depending on data distribution. Categorical data were presented as frequencies and corresponding percentages. Categorical and non-normally distributed continuous data were analyzed using the Pearson *χ*^2^ test, the Fisher exact test, or the Kruskal–Wallis test, as deemed appropriate. *P* < 0.05 indicated statistically significant differences. Covariate adjustments were applied where any of the following criteria were met: (1) confounders reported in the literature, (2) univariate analysis yielding a *P*-value of <0.1, or (3) a change in effect size of >10% upon covariate inclusion. Comprehensive univariate regression analysis was conducted on all variables to ascertain the potential factors that predict primary outcomes (adverse short-term neonatal effects). Univariate analysis was used to reveal the trends associated with adverse short-term neonatal outcomes. Logistic regression analysis was used to assess the independent association between dexamethasone exposure and adverse short-term neonatal effects. Subgroup analyses by age, parity, gravidity, and common comorbidities, such as pregnancy-induced hypertension (PIH) and gestational diabetes mellitus (GDM), were used to examine the stability of the association between dexamethasone exposure and adverse short-term neonatal effects. Smooth curve-fitting and threshold saturation effect analyses were used to assess the probability of short-term adverse neonatal effects. The likelihood of these effects was quantified using odds ratios (OR) and standard error with 95% confidence intervals (CI). Statistical analyses were done on R (http://www.R-project.org) and Free Statistics software version 1.8. A two-tailed test was employed, *P* < 0.05 indicating statistically significant differences.

## 3 Results

### 3.1 Baseline characteristics

The baseline characteristics of the study's participants based on dexamethasone treatment status are shown in Table 1. From the original dataset of 560 observations, 17 entries were excluded because of a lack of crucial covariate information. Of these, 12 were excluded because of missing Apgar score data at one minute, one because of missing Apgar score data at 10 min, two because of missing gravidity data, and two because of missing GDM and PIH data. Hence, the final analysis involved 543 women and their neonates. The women had an average age of 32.3 ± 4.5 years and the majority (95.4%) identified as Sinhalese. Of the participants, 121 (22.2%) underwent planned cesarean section and received dexamethasone before the procedure (Table 1). The fetal growth restriction (FGR) rate was 5% more prevalent in the non-dexamethasone-treated group compared to a 0.8% lower incidence in the dexamethasone-treated group (*P* = 0.038). However, various factors, such as reproductive history (gravidity, parity, children), pregnancy comorbidities (GDM, polyhydramnios, and PIH), gestational age at cesarean section, and important neonatal characteristics like neonatal weight and Apgar scores at 1, 5, and 10 min) did not differ significantly between the two groups (*P* > 0.05).

**Table 1:** Baseline characteristics of study patients.

Variables	Total	Dexamethasone group	Control group	*P-*value
	*n* = 543	*n* = 121	*n* = 422	
Age, mean (SD), years	32.3 ± 4.5	32.4 ± 4.5	32.3 ± 4.5	0.827
Ethnicity, *n* (%)				0.127
Sinhalese	518 (95.4)	113 (93.4)	405 (96)	
Tamil	10 (1.8)	4 (3.3)	6 (1.4)	
Muslim	12 (2.2)	2 (1.7)	10 (2.4)	
Other	3 (0.6)	2 (1.7)	1 (0.2)	
Gravidity,mean (SD)	2.3 ± 1.0	2.3 ± 1.1	2.3 ± 1.0	0.659
Parity, Median (IQR)	2.0 (1.0, 2.0)	2.0 (1.0, 2.0)	2.0 (1.0, 2.0)	0.763
Children, Median (IQR)	1.0 (0.0, 1.0)	1.0 (0.0, 1.0)	1.0 (0.0, 1.0)	0.794
GDM, *n* (%)	93 (17.1)	15 (12.4)	78 (18.5)	0.117
Polyhydramnios, *n* (%)	2 (0.4)	0 (0)	2 (0.5)	1
PIH, *n* (%)	54 (9.9)	8 (6.6)	46 (10.9)	0.165
FGR, *n* (%)	22 (4.1)	1 (0.8)	21 (5)	0.038
Gestational age at cesarean section,mean (SD), days	265.9 ± 3.7	265.9 ± 4.1	265.9 ± 3.5	0.887
Birth weight,mean (SD), kg	2.9 ± 0.4	3.0 ± 0.5	2.9 ± 0.4	0.276
Apgar score at 1 min, mean (SD)	9.5 ± 0.7	9.5 ± 0.6	9.4 ± 0.7	0.32
Apgar score at 5 min, mean (SD)	10.0 ± 0.1	10.0 ± 0.2	10.0 ± 0.1	0.397
Apgar score at 10 min, mean (SD)	10.0 ± 0.0	10.0 ± 0.0	10.0 ± 0.0	0.593
Short-term adverse neonatal outcomes, *n* (%)	156 (28.7)	87 (71.9)	69 (16.4)	<0.001
NICU admission, *n* (%)	5 (0.9)	2 (1.7)	3 (0.7)	0.31
SCBU admission, *n* (%)	12 (2.2)	4 (3.3)	8 (1.9)	0.315
Diagnosis of TTN, *n* (%)	26 (4.8)	13 (10.7)	13 (3.1)	<0.001
Diagnosis of RDS, *n* (%)	21 (3.9)	9 (7.4)	12 (2.8)	0.03
Intravenous Antibiotics, *n* (%)	147 (27.1)	86 (71.1)	61 (14.5)	<0.001
Ventilator support, *n* (%)	5 (0.9)	4 (3.3)	1 (0.2)	0.01
Days of hospital stay, Median (IQR), days	1.0 (1.0, 2.0)	2.0 (2.0, 2.0)	1.0 (1.0, 2.0)	<0.001

SD, standard deviation; IQR, interquartile range; GDM, gestational diabetes mellitus; PIH, pregnancy-induced hypertension; FGR, fetal growth restriction; NICU, neonatal intensive care unit; SCBU, special care baby unit; TTN, transient tachypnoea of newborn; RDS, respiratory distress syndrome.

The dexamethasone-treated cohort had significantly higher rates of primary outcome measures, such as transient neonatal tachypnea, respiratory distress syndrome, intravenous antibiotic administration, ventilatory support, and the duration of neonatal hospitalization when compared with the control group (*P* < 0.05). Moreover, in the dexamethasone group, the probabilities of neonatal admission into the NICU or placement in the special care baby unit were 1.7% and 3.3%, respectively, which was higher than in the control group.

This study observed a notable disparity in the utilization of antibiotics between the groups exposed to dexamethasone and those not exposed. A review of existing literature indicated varying rates of antibiotic usage for newborns admitted to neonatal intensive care units (NICUs), ranging from 2.4% to 97.1% (27). In order to address this issue, logistic single-factor analysis was conducted, and adjustments were made to the effect size if it exceeded a 10% change upon the inclusion of covariates. Potential confounding factors that were taken into consideration included variations in Admission to Neonatal Intensive Care Unit, Documented Transient Tachypnea of Newborn, and Documented Respiratory Distress Syndrome between the two groups (Supplementary Table S3). Furthermore, owing to the higher occurrence of short-term adverse outcomes in the dexamethasone-treated group, there was an increased likelihood of admission to the Neonatal Intensive Care Unit and diagnoses of Documented Transient Tachypnea of Newborn and Documented Respiratory Distress Syndrome, thereby contributing to the escalated usage of antibiotics in the dexamethasone group.

### 3.2 Association between dexamethasone and short-term adverse neonatal outcomes in full-term elective cesarean delivery

Univariate analysis revealed statistically significant associations between short-term adverse neonatal outcomes and parity (OR: 0.74, 95% CI: 0.60–0.92, *P* = 0.006), 5-minute Apgar scores (OR: 0.11, 95% CI: 0.01–0.86, *P* = 0.035), time interval between dexamethasone administration and cesarean section (OR: 0.16, 95% CI: 0.06–0.46, *P* = 0.001) and neonatal duration of hospital stay (OR: 4.79, 95% CI: 3.42–6.71, *P* < 0.001) (Table 2). Multiple logistic regression results of the analysis of the association between dexamethasone and short-term adverse neonatal outcomes are presented in Table 3. The unadjusted model (Model I) showed that dexamethasone was significantly associated with an increased risk of short-term adverse neonatal outcomes (OR: 13.09, 95% CI: 8.16–21.01, *P* < 0.001), and the association remained significant after adjusting for age, ethnicity, gravidity, parity, and children (Model II; OR: 16.46, 95% CI: 9.81–27.64, *P* < 0.001). Moreover, this association did not change significantly after adjustment for GDM, PIH, and FGR (Model III; OR: 17.34, 95% CI: 10.21–29.47, *P* < 0.001), or all other relevant comorbidities, including birth weight, 1- and 5-minute Apgar scores, duration of hospital stay, and gestational age at cesarean section (Model IV; OR: 12.76, 95% CI: 6.9–23.62, *P* < 0.001).

**Table 2:** Association of covariates dexamethasone and short-term adverse neonatal outcomes risk.

Variable	OR:, 95% CI	*P*-value
Age, years	1.01 (0.97–1.05)	0.699
Ethnicity		
Sinhalese	1 (Reference)	
Tamil	1.07 (0.27–4.2)	0.921
Muslim	0.5 (0.11–2.31)	0.375
Gravidity	1 (0.84–1.2)	0.966
Parity	0.74 (0.6–0.92)	0.006
Children	1.14 (0.89–1.46)	0.301
GDM: No vs. Yes	1.47 (0.87–2.49)	0.152
PIH: No vs. Yes	1.05 (0.56–1.97)	0.871
FGR: No vs. Yes	1.39 (0.5–3.83)	0.527
Gestational age at cesarean section, days	1.02 (0.96–1.07)	0.559
Time interval between dexamethasone administration and cesarean section, days	0.16 (0.06–0.46)	0.001
Birth weight, kg	1.2 (0.77–1.86)	0.427
Apgar score at 1 min	0.92 (0.7–1.2)	0.524
Apgar score at 5 min	0.11 (0.01–0.86)	0.035
Days of Hospital Stay, days	4.79 (3.42–6.71)	<0.001

GDM, gestational diabetes mellitus; PIH, pregnancy-induced hypertension; FGR, fetal growth restriction.

**Table 3:** Multivariate regression analysis of the association between dexamethasone and short-term adverse neonatal outcomes.

Variable	Control group	Dexamethasone group	*P*-value
	OR (95% CI)	OR (95% CI)	
Model 1^a^	1 (Ref)	13.09 (8.16–21.01)	<0.001
Model 2^b^	1 (Ref)	16.46 (9.81–27.64)	<0.001
Model 3^c^	1 (Ref)	17.34 (10.21–29.47)	<0.001
Model 4^d^	1 (Ref)	12.76 (6.9–23.62)	<0.001

^a^ Model 1: No adjusted.

^b^ Model 2: Adjusted for age + ethnicity + gravidity + parity + children.

^c^ Model 3: Model 2 + GDM + PIH + FGR.

^d^ Model 4: Model 3 + birth weight + apgar score at 1 min + apgar score at 5 min + days of hospital stay + gestational age at cesarean section.

### 3.3 Sensitivity and subgroup analysis

**Figure 1:**
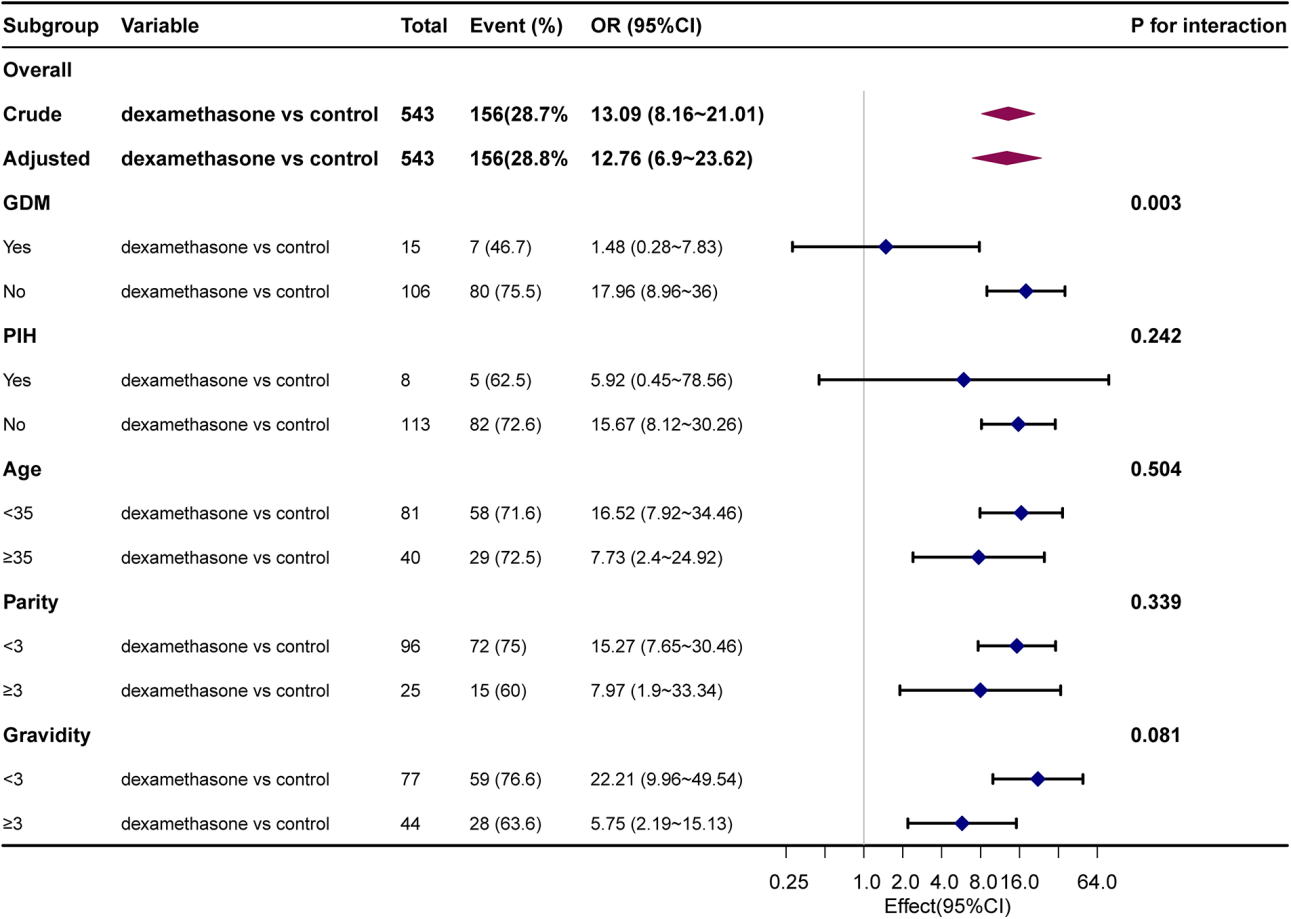
Association between dexamethasone and short-term adverse neonatal outcomes. Adjusted for ethnicity, children, GDM, birth weight, Apgar at one and five minutes, days of hospital stay, and gestational age at cesarean section.

**Figure 2:**
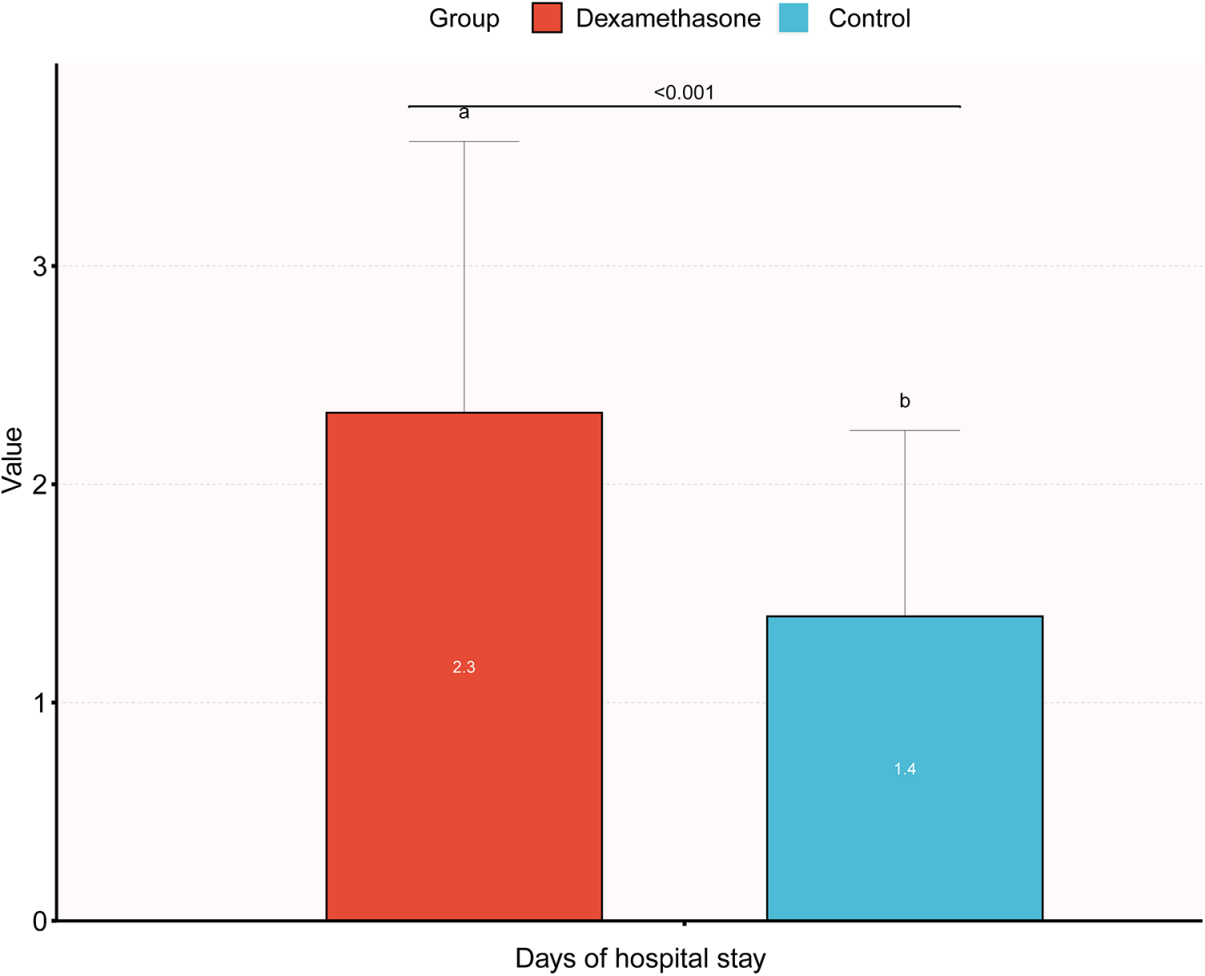
Bar chart of the duration of hospitalization in the dexamethasone and control groups.

The results of stratified and interaction analyses of dexamethasone and short-term adverse neonatal outcomes subgroups of key factors were analyzed in stratified and interaction analyses. The stratified analysis showed that PIH, age, parity, and gravidity were not statistically significant after stratification (*P* > 0.05), indicating that the effect of dexamethasone on short-term adverse neonatal outcomes was stable and it was not affected by changes in covariates (Figure 1). The association between GDM and dexamethasone was examined for short-term adverse neonatal outcomes (*P* = 0.003). There is an increased likelihood that dexamethasone is linked to short-term adverse pregnancy outcomes in neonates whose mothers did not have gestational diabetes before pregnancy when compared with the control group. A comprehensive analysis of the correlation between the duration of neonatal hospitalization and dexamethasone, focusing on immediate negative consequences revealed a significant increase in the average length of hospitalization for neonates in the dexamethasone group when compared with the control group, indicating a comparatively more critical condition. Notably, the difference in the mean hospital stay between the two groups was only 0.9 days, with the maximum hospital stay being 12 days and there were no neonatal mortalities. This suggests that dexamethasone may have a transient effect on short-term adverse neonatal outcomes with a better overall prognosis (Figure 2).

## 4 Discussion

This study explored the association between dexamethasone usage during full-term elective cesarean deliveries and short-term adverse neonatal outcomes. This single-center retrospective cohort study made the following key findings: (1) there was a significantly elevated likelihood of short-term adverse neonatal outcomes in the cohort that received dexamethasone before full-term planned cesarean sections, (2) dexamethasone was significantly associated with short-term adverse neonatal outcomes, even after adjusting for baseline characteristics and other comorbidities, and (3) the possible effects of dexamethasone on short-term adverse neonatal outcomes are transient. Prenatal corticosteroid therapy, a fundamental component of perinatal care, warrants comprehensive evaluation because of its potential impact on fetal development and programming, given its influence on up to 20% of the transcriptome (28, 29). To avert adverse short-term neonatal outcomes it is important to minimize fetal exposure to such medications. Notably, full-term and late preterm infants are inherently exposed to elevated levels of endogenous steroids and may additionally receive prenatal corticosteroids (30). A recent meta-analysis involving 1.6 million infants found that early prenatal exposure to corticosteroids, when compared to no direction, was associated with an increased risk of neonatal intensive care unit admissions among full-term infants (OR: 1.49, 95% CI: 1.19–1.86) (23). This highlights the pressing need for informed, evidence-based use of corticosteroids to minimize the potential for over-treatment and subsequent neonatal mortality.

Furthermore, previous studies indicate that dexamethasone administration during pregnancy has potential adverse consequences, including an increased risk of cardiovascular disease in the offspring and neurotoxicity (31–35).

Our study revealed a significant correlation between dexamethasone usage and short-term adverse neonatal outcomes in full-term elective cesarean deliveries, which persisted even after adjusting for covariates linked to short-term neonatal adverse effects. Specifically, neonates in the dexamethasone-treated group exhibited a higher risk of various unfavorable outcomes, including respiratory distress syndrome, intravenous antibiotic use, ventilatory support, and a greater likelihood of admission into neonatal intensive care. These findings raise concerns about the potential risks associated with dexamethasone, particularly during full-term elective cesarean deliveries. Countries must exercise prudence when incorporating interventions into healthcare policies and ensure that decisions are rooted in evidence of efficacy and a thorough risk assessment (36).

However several studies with similar aims found that prenatal corticosteroid treatment did not clearly establish a significant association with adverse neonatal outcomes (11, 12, 37). Although these studies are relevant to our topic, they differ in their primary outcome indicators. Specifically, Stutchfield et al. (12) focused on the incidence of neonates admitted to the intensive care nursery for respiratory distress, whereas our study focused on neonates requiring specialized care as the outcome indicator. This disparity could potentially serve as a significant determinant influencing the disparate findings observed in our study. Concurrently, it is worth acknowledging that the systematic review conducted by Sotiriadis et al. (11) exhibits certain uncertainties regarding the precision of its findings, owing to the limitations associated with the certainty of evidence available in the existing literature. Conversely, our study primarily classified unfavorable outcomes based on the requirement for specialized medical attention, with a specific emphasis on the potential detrimental impacts of medications on neonatal outcomes. While acknowledging the potential divergence from existing literature, it is our firm conviction that our study contributes significant insights into distinct neonatal outcomes.

Our study found that the effect of dexamethasone on short-term adverse neonatal outcomes was transient. Specifically, although the dexamethasone group was associated with an increased risk of adverse neonatal outcomes, this effect was mainly manifested in the short term and did not persist over an extended period. No neonatal deaths were observed despite a prolonged duration (days) of hospitalization, and the mean difference was relatively small. A retrospective cohort study involving data from 588,077 live births found that antenatal corticosteroid exposure was associated with significantly lower odds of neonatal mortality and 5-minute Apgar scores of <7 in 121,151 women (38). However, there was an increased incidence of some adverse neonatal outcomes, such as surfactant replacement therapy, prolonged mechanical ventilation, antibiotics for suspected neonatal sepsis, and NICU admissions. The causes may include lung maturation and the anti-inflammatory effects of prenatal corticosteroids, which may enhance alveolar complexity (39), and the immunosuppressive effects of corticosteroids, which may cause or worsen infections (40). However, further studies are needed to confirm the long-term neuropsychiatric and cardiac risks.

### 4.1 Limitations of the study and suggestions for future research

(1) Data Source and Confounders: The present study relied on secondary data obtained from previous research, thereby imposing inherent constraints on the availability of information about potential confounding variables. (2) Study design: Being retrospective, this investigation is susceptible to inherent limitations, such as the potential existence of unmeasured confounders. Furthermore, the observational nature of the study does not establish causality but only offers evidence of association. The single-center nature of the study may limit the generalizability of its findings. (3) Based on the admission records, this retrospective cohort study identified one case of neonatal hypoglycemia among the hospitalized newborns. However, postnatal neonatal blood glucose data were not collected.

To address these limitations and advance our understanding of dexamethasone usage during full-term elective cesarean deliveries, future research should consider the following: (1) performing multicenter prospective studies, which can enable a more comprehensive and robust examination of the role of dexamethasone in full-term elective cesarean deliveries. These studies should include a wider range of patient characteristics and factors to better evaluate the impact of dexamethasone. (2) Future research should delve deeper into the timing of dexamethasone administration and how patient characteristics may influence its outcomes. This will help elucidate the nuances of dexamethasone use in different clinical scenarios. (3) The impact of prenatal dexamethasone on maternal well-being, including conditions like hyperglycemia and hypertension also warrants investigation. Furthermore, it is important to examine the potential of enduring consequences of prenatal steroid exposure on childhood development (41), including neurodevelopmental outcomes and related factors.

Addressing these limitations and conducting further research can improve our understanding of the benefits and potential risks of dexamethasone in the context of full-term elective cesarean deliveries.

## 5 Conclusion

Despite our study's limitations, our findings indicate a possible correlation between dexamethasone administration during elective cesarean delivery at full term and negative neonatal outcomes in the short term. However, these findings require further validation through thorough investigations. Nonetheless, healthcare professionals should exercise caution when considering dexamethasone therapy and carefully evaluate the trade-off between potential advantages and risks, while engaging in shared decision-making with patients to determine the most appropriate treatment strategy.

## Data Availability

The datasets presented in this study can be found in online repositories. The names of the repository/repositories and accession number(s) can be found below: https://doi.org/10.5061/dryad.g79cnp5qs.

## Supplementary material

Supplementary Figure S1 Graphs showing smooth curve fittings of the gestation period at cesarean section and short-term adverse neonatal outcomes. Abbreviations: The solid and dashed lines represent the estimated values and their corresponding 95% confidence intervals. Analysis was adjusted for age, ethnicity, gravidity, parity, children, GDM, PIH, FGR, birth weight, and Apgar at one and five minutes. The solid and dashed lines represent the predicted value and corresponding 95% confidence intervals. The density curve of gestational age distribution during cesarean section is illustrated by the blue region. The median gestational age at cesarean section is represented by the vertical dashed line at 266 days in the legend.

Supplementary Table S1 Threshold effect analysis.

Supplementary Table S2 Collinear diagnosis.
