# Supplementary figures and images for: The influence of prenatal dexamethasone administration before scheduled full-term cesarean delivery on short-term adverse neonatal outcomes: a retrospective single-center cohort study
Source: Front Pediatr. 2024 Jan 11;11:1323097. doi: 10.3389/fped.2023.1323097 (PMC10808727; doi:10.3389/fped.2023.1323097)

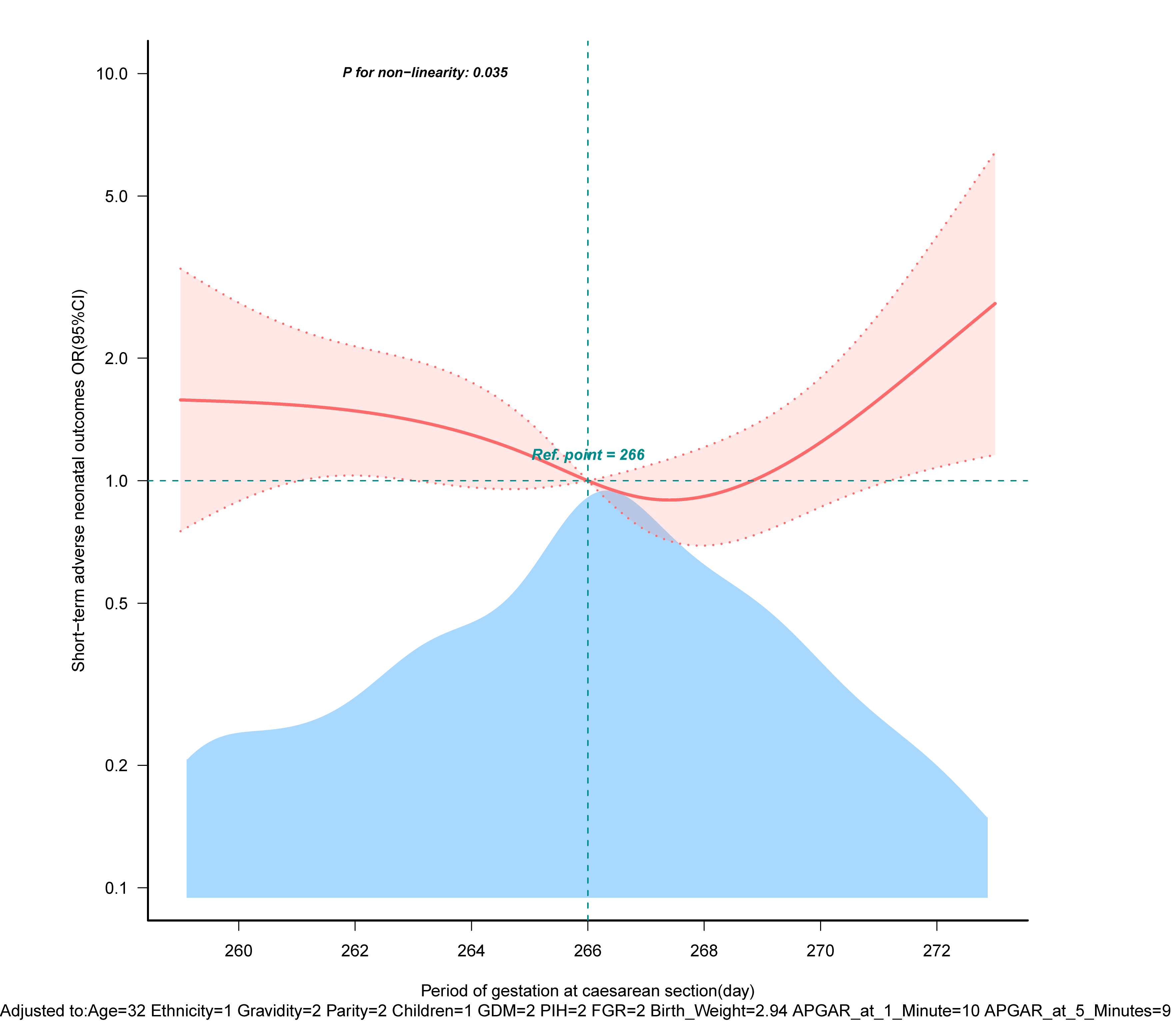

Supplement: Supplementary Figure S1 — Graphs showing smooth curve fittings of the gestation period at cesarean section and short-term adverse neonatal outcomes. Abbreviations: The solid and dashed lines represent the estimated values and their corresponding 95% confidence intervals. Analysis was adjusted for age, ethnicity, gravidity, parity, children, GDM, PIH, FGR, birth weight, and Apgar at one and five minutes. The solid and dashed lines represent the predicted value and corresponding 95% confidence intervals. The density curve of gestational age distribution during cesarean section is illustrated by the blue region. The median gestational age at cesarean section is represented by the vertical dashed line at 266 days in the legend. [file Image1.tif]
