# Supplementary material for: The influence of prenatal dexamethasone administration before scheduled full-term cesarean delivery on short-term adverse neonatal outcomes: a retrospective single-center cohort study
Source: Front Pediatr. 2024 Jan 11;11:1323097. doi: 10.3389/fped.2023.1323097 (PMC10808727; doi:10.3389/fped.2023.1323097)
Supplement: Supplementary Table S1 — Threshold effect analysis. [file Table1.doc]

# Table S1 Threshold effect analysis of the gestation age at cesarean section on short-term adverse neonatal outcomes using the two-piecewise linear regression model.

| **Short-term adverse neonatal outcomes** | **OR (95% CI)** | *P****-value*** |
| --- | --- | --- |
| **Standard linear model** | 1 (0.94~1.05) | 0.9 |
| ****Two-piecewise linear model**** |  |  |
| **Inflection point** | 269 |  |
| **259 ≤ Gestational age at cesarean section <269 **(days)**** | 0.97 (0.89~1.06) | 0.498 |
| **269 ≤ Gestational age at cesarean section≤273 **(days)**** | 1.69 (1.21~2.36) | 0.002 |
| **Log likelihood ratio** |  | 0.001 |

Age was converted into exposure variable. Age, Ethnicity, Gravidity, Parity, Children, GDM, PIH, FGR, Birth Weight, APGAR at 1 Minutes and APGAR at 5 Minutes were adjusted.
