# Supplementary material for: The influence of prenatal dexamethasone administration before scheduled full-term cesarean delivery on short-term adverse neonatal outcomes: a retrospective single-center cohort study
Source: Front Pediatr. 2024 Jan 11;11:1323097. doi: 10.3389/fped.2023.1323097 (PMC10808727; doi:10.3389/fped.2023.1323097)
Supplement: Supplementary Table S2 — Collinear diagnosis. [file Table2.doc]

**Table S3 Association of covariates dexamethasone and intravenous antibiotics risk**

| **Variable** | **OR_95CI** | ***VIF*** | ***P-value*** |
| --- | --- | --- | --- |
| **Age, years** | 1.01 (0.97~1.05) |  | 0.716 |
| **Ethnicity** |  |  |  |
| Sinhalese | 1 (Reference) |  |  |
| Tamil | 1.17 (0.3~4.58) |  | 0.823 |
| Muslim | 0.55 (0.12~2.52) |  | 0.437 |
| **Gravidity** | 0.98 (0.82~1.18) |  | 0.855 |
| **Parity** | 0.69 (0.55~0.86) | 1.091 | **0.001** |
| **Children** | 1.08 (0.84~1.39) |  | 0.549 |
| **GDM:No vs Yes** | 1.44 (0.84~2.45) |  | 0.186 |
| **PIH:No vs Yes** | 1.33 (0.68~2.61) |  | 0.399 |
| **FGR:No vs Yes** | 2.42 (0.71~8.3) |  | 0.16 |
| **Gestational age at cesarean section,days** | 1.02 (0.97~1.08) |  | 0.389 |
| **Birth weight,kg** | 1.37 (0.88~2.16) |  | 0.168 |
| **Apgar score at 1 minutes** | 1.06 (0.79~1.41) |  | 0.711 |
| **Apgar score at 5 minutes** | 0.35 (0.1~1.23) | 1.222 | 0.102 |
| **Days of Hospital Stay,days** | 4.69 (3.35~6.57) | 1.278 | **<0.001** |
| **NICU admission** | 11.05 (1.22~99.68) | 1.005 | **0.032** |
| **Diagnosis of TTN** | 13.03 (4.81~35.28) | 1.25 | **<0.001** |
| **Diagnosis of RDS** | 18.28 (5.3~63.06) | 1.046 | **<0.001** |

Abbreviations: GDM, gestational diabetes mellitus; PIH, pregnancy-induced hypertension; FGR, fetal growth restriction; NICU, Neonatal intensive care unit; TTN, transient tachypnoea of newborn; RDS, respiratory distress syndrome.
